# SNHG15 knockdown inhibits diabetic nephropathy progression in pediatric patients by regulating the miR-141/ICAM-1 axis *in vitro*

**DOI:** 10.1042/BSR20204099

**Published:** 2021-02-08

**Authors:** Jiewei Liu, Dongliang Cai, Ying Wang, Yanhong Zou, Tana Zhao

**Affiliations:** 1 The Second Department of Pediatrics, The First Affiliated Hospital of Jiamusi University, No. 238, Dexiang Street, Jiamusi City, Heilongjiang Province 154002, China; 2 Department of Geratology, The First Affiliated Hospital of Jiamusi University, No. 238, Dexiang Street, Jiamusi City, Heilongjiang Province 154002, China

**Keywords:** diabetic nephropathy, high-glucose, ICAM-1, lncRNA SNHG15, miR-141

## Abstract

Long non-coding RNAs (lncRNAs) are confirmed to be involved in modulating diabetic nephropathy (DN). The present study is aimed to explore the regulatory mechanism of lncRNA small nucleolar RNA host gene 15 (SNHG15) on pediatric DN. Human glomerular mesangial cells (HGMCs) were exposed to high glucose (HG) to produce an *in vitro* model. The results showed that SNHG15 was remarkably up-regulated in pediatric DN tissues and HG-induced HGMCs. Functional experiments indicated that both silencing of SNHG15 and overexpression of miR-141 elevated the cell viability, and suppressed the inflammation in HG-induced HGMCs. SNHG15 was identified to be a lncRNA that could bind to miR-141, and ICAM-1 was a downstream target gene of miR-141. Both the low expression of miR-141 and high expression of ICAM-1 reversed the inhibiting effect of SNHG15 knockdown on inflammatory response, and the promoting effect on cell viability. To conclude, our study revealed that silencing of SNHG15 ameliorated the malignant behaviors of pediatric DN via modulating the miR-141/ICAM-1 axis *in vitro*.

## Introduction

Diabetic nephropathy (DN) is a main and serious complication of diabetes mellitus (DM) [1,2]. The increasing rates of childhood obesity worldwide have been reported to be strongly associated with the rising prevalence of DM [3]. In especial, microalbuminuria (MA) or incipient DN is the most common abnormal finding in diabetic children and adolescents, whereas overt proteinuria is found in less than 1–1.5% of pediatric patients [4,5]. The pathological features of DN are in many ways and inflammation is one of the most common pathological factors [6,7]. Numerous researches have indicated that inflammation response in renal facilitates the development of DN [8,9]. Although various therapies such as controlling blood glucose [10] and inflammation [11] for treating DN have been improved, the therapeutic effects remain disillusionary. Therefore, further molecular research on the potential mechanism of DN is urgent to be solved to develop promising therapeutic targets in DN.

Some long non-coding RNAs (lncRNAs) are widely known to be participated in modulating cellular processes, including release of inflammatory cytokines in an *in vitro* model of DN [12–14]. Zhang et al. have disclosed that knockdown of lncRNA Rpph1 restrains inflammatory cytokines release in mice mesangial cells (MMCs) [12]. Peng et al. have uncovered the decreased inflammatory-factors levels of mesangial cells (MCs) were positively correlated with lncRNA NONHSAG053901 down-regulation [13]. Ma et al. have illustrated that inflammatory reaction in HG-induced MMCs was suppressed by silencing of lncRNA NEAT1 [14]. The above researches indicated that some lncRNAs serve as a proinflammatory-factor role in cells. Interestingly, small nucleolar RNA host gene 15 (SNHG15) has been reported to serve as a morbigenous lncRNA in human cancers, such as lung cancer [15], breast cancer [16], ovarian cancer [17] and prostate cancer [18]. More importantly, also as a member of SNHG family, SNHG16 has been confirmed to play a promoting role in the proliferation of HG-induced MMCs and fibrosis of DN tissues [19]. However, whether SNHG15 is involved in modulating inflammatory response and the development of DN remains unclear.

Increasing researches have been demonstrated that microRNAs (miRNAs) exhibit their suppressive roles in DN progression or cell inflammation [20–22]. For instance, miR-181a overexpression declines concentrations of IL-6 and IL-1β in mesangial cells, eventually alleviating the inflammatory response of DN [20]. MiR-218 up-regulation visibly restrains IL-6, IL-1β and TNF-α levels of murine podocyte cells [21]. Overexpression of miR-485 dampens the inflammation and proliferation of mesangial cells in an in vitro model of DN [22]. Of note, miR-141 has also been reported to act as a suppressor on DN *in vitro* [19,23]. Recent studies have revealed that the increased miR-141 expression significantly attenuates renal fibrosis [19,23] and cell proliferation [19]. However, the effect of miR-141 on inflammation of MCs and the regulatory mechanism between miR-141 and SNHG15 have not been fully elucidated.

In the present study, we investigated the effects of lncRNA SNHG15 on the viability and inflammation of HGMCs, and explored the regulatory mechanisms between SNHG15 and miR-141/ICAM-1 on pediatric DN. The present study highlighted the molecular mechanism of how SNHG15 mediates the progression of DN *in vitro*.

## Methods

### Tissues collection

From 2017 to 2018, totally 15 pediatric DN patients without other complications were selected in our hospital. DN patients had not received treatment within 3 months prior to admission. Simultaneously, 20 healthy children undergone a physical examination were recruited as controls. The pathological kidney tissues of the patients and the normal kidney tissues of the healthy children were obtained by biopsy. Written informed consent was obtained for each patient. The present study obtained the approval of the Ethics Committee in our hospital.

### Cell culture, grouping and transfection

Human glomerular mesangial cells (HGMCs) were purchased from Xinyu Biotech, Ltd (Shanghai, China) and cultured in DMEM containing 10% fetal bovine serum (FBS) at 37°C. Then, HGMCs were further divided into two groups: the high glucose (HG, 33.3 mM glucose) group and normal glucose (NG, 5.5 mM glucose) group.

ShRNA-negative control (sh-NC) and shRNA-SNHG15 (sh-SNHG15) were procured from Sangon Biotech, Inc (Shanghai, China). MiR-141 inhibitor and its negative control inhibitor NC, miR-141 mimics and its negative control miR-NC, overexpression-SNHG15 (oe-SNHG15), overexpression-ICAM-1 (oe-ICAM-1) and their negative control pcDNA3.1, were all procured from Ribo Biotech, Ltd (Guangzhou, China). HG-induced HGMCs were transfected with the aforementioned agents using Lipofectamine RNAiMAX kit (Invitrogen, Carlsbad, CA, U.S.A.) for 48 h. Subsequently, the cells were harvested to perform the following experiments.

### Quantitative reverse-transcription PCR (qRT-PCR)

Following the manufacturer’s instructions, total RNA from pediatric DN tissues or HG-induced HGMCs was extracted using a TRIzol kit (Invitrogen, Inc.). The extracted RNA was reverse transcribed into cDNA using the GoScript reverse transcription system (Promega, Madison, WI, U.S.A.) and then subjected to qRT-PCR analyses. The thermocycling conditions were: 95°C for 10 min, followed by 40 cycles of 95°C for 10 s, 60°C for 20 s and 72°C for 34 s. GAPDH or U6 was used as the internal reference. Gene expression was quantified using the 2^−ΔΔCt^ method.

### MTT assay

The HG-induced HGMCs cells were seeded into a 96-well plate with 2 × 10^5^ cells per well at 37°C. After incubation for 24 h, 20 µl MTT (GENECHEM, Inc, Shanghai, China) was added to each well to incubate for another 2 h at 37°C. The viability (OD450) was analyzed using a Multiskan Spectrum microplate reader (Thermo Fisher Scientific, Waltham, MA, U.S.A.).

### Enzyme-linked immunosorbent assay (ELISA)

The levels of the inflammatory cytokines (TNF-α, IL-1β and IL-6) in HG-induced HGMCs were measured using a specific ELISA Kit (Multisciences Biotech, Ltd., Hangzhou, China) in accordance with the manufacturer protocol. A Multiskan Spectrum microplate reader (Thermo Fisher Scientific) was used to determine the absorbance at 450 nm.

### Dual luciferase reporter (DLR) assay

SNHG15 with WT or MUT miR-141-binding sites were fused to the pGL3 vector. The 3’-UTR of ICAM-1 containing the predicted miR-141 targeting site (WT) and MUT ICAM-1 were cloned into the pGL3 vector. Wild-type/mutant-type vector and miR-141 mimics/miR-NC were co-transfected into HGMCs for 48 h at 37°C. The relative luciferase activity was detected using a Dual-Luciferase Reporter Assay System (Promega).

### Western blot analysis

The proteins from HG-induced HGMCs were extracted using RIPA buffer containing protease inhibitor, followed by detecting the protein concentrations by a BCA Protein Assay Kit (Abcam). Then, proteins were separated by 10% SDS-PAGE and transferred into PVDF membrane. At room temperature, the blocking was performed using 5% bovine serum albumin (BSA). After that, the membrane was incubated with primary antibodies against ICAM-1 (1:1000; Abcam) and β-actin (1:1000; Abcam) for overnight at 4°C. Then, tris-buffered saline Tween-20 (TBST) was used to wash the membrane for three times. Subsequently, at room temperature, the secondary antibody HRP-conjugated anti-mice IgG (1:5000; Santa Cruz, Waltham, MA, U.S.A.) was added to incubate for 1 h. β-Actin was used as the internal reference. The membrane was developed by Chemiluminescence reagents (Thermo Fisher Scientific) under Gel-Pro analyzer (version 4.0, U.S.A.).

### Statistical analysis

SPSS software (version 20.0, U.S.A.) was used to perform statistical analyses. Data were expressed as the mean ± standard deviation. Student’s *t*-test was utilized to assess the differences between two groups, whereas one-way ANOVA was used to evaluate the differences among multiple groups. After ANOVA analysis, the pairwise comparison was performed using Tukey’s multiple comparisons test. *P*-value less than 0.05 indicated a statistically significant difference. All experiments were conducted in triplicate in at least three independent experiments.

## Results

### Release of inflammatory cytokines in HG-induced HGMCs is restrained by SNHG15 knockdown

**Figure 1:**
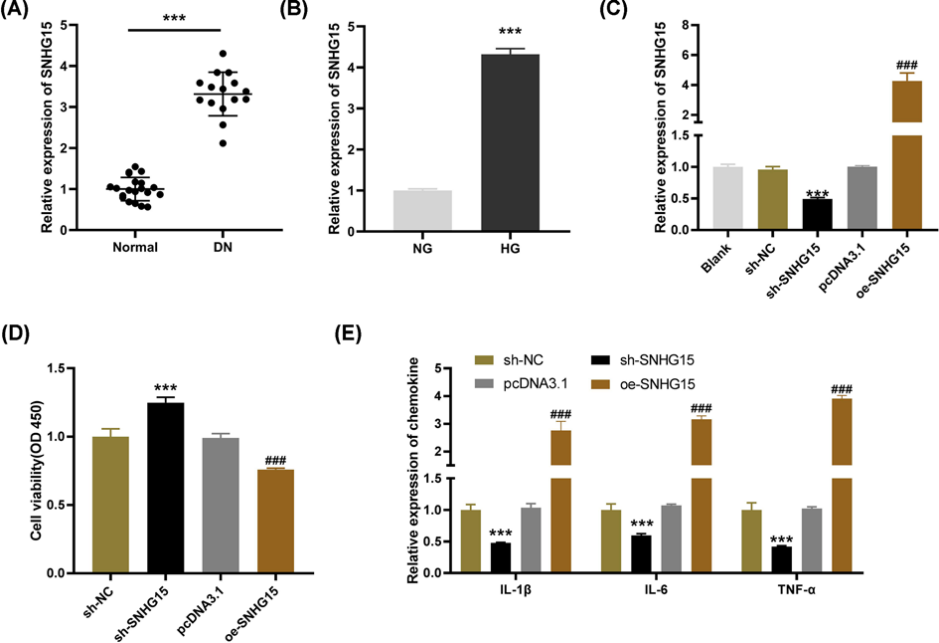
Release of inflammatory factors in high-glucose (HG)-induced human glomerular mesangial cells (HGMCs) is restrained by small nucleolar RNA host gene 15 (SNHG15) knockdown (**A**) The expression of SNHG15 in diabetic nephropathy (DN) tissues (*n*=15) and normal tissues (*n*=20) was detected by quantitative reverse-transcription PCR (qRT-PCR); ***P<0.001 vs*.* normal. (**B**) The expression of SNHG15 in HG-stimulated HGMCs was determined by qRT-PCR; ***P<0.001 vs*.* the normal glucose (NG) group. (**C**) The expression of SNHG15 after transfection of shRNA (sh)-SNHG15/negative control (NC) or overexpression (oe)-SNHG15/pcDNA3.1 into HG-stimulated HGMCs was detected by qRT-PCR. (**D**) The viability of HG-stimulated HGMCs was measured by MTT assay. (**E**) The levels of IL-1β, IL-6 and TNF-α in HG-stimulated HGMCs were measured by ELISA. ***P<0.001 vs*.* the sh-NC group; ### *P*<0.001 vs*.* the pcDNA3.1 group.

SNHG15 expression in pediatric DN and normal tissues was detected by qRT-PCR. The results uncovered that the expression of SNHG1 in pediatric DN tissues was up-regulated in contrast with that in normal tissues (Figure 1A, *P*<0.001). Meanwhile, we found that SNHG15 expression was also increased in HG-induced HGMCs compared with the NG controls. (Figure 1B, *P*<0.001). After transfection of sh-SNHG15/NC or pDNA3.1/oe-SNHG15 into HG-stimulated HGMCs, the efficiency of transfection was detected. The results of qRT-PCR revealed that SNHG15 expression was visibly down-regulated by sh-SNHG15 and up-regulated by oe-SNHG15 (Figure 1C, *P*<0.001). As shown in Figure 1D, the viability of HG-induced HGMCs was promoted by transfection of sh-SNHG15, whereas was inhibited by transfection of oe-SNHG15 (*P*<0.001). Interestingly, the opposite results were obtained in the levels of inflammatory cytokines. Through ELISA, we discovered that SNHG15 knockdown significantly decreased and SNHG15 overexpression elevated the levels of IL-6, IL-1β and TNF-α (Figure 1E, *P*<0.001). These results indicated that SNHG15 might be a proinflammatory factor in DN.

### LncRNA SNHG15 targets miR-141

**Figure 2:**
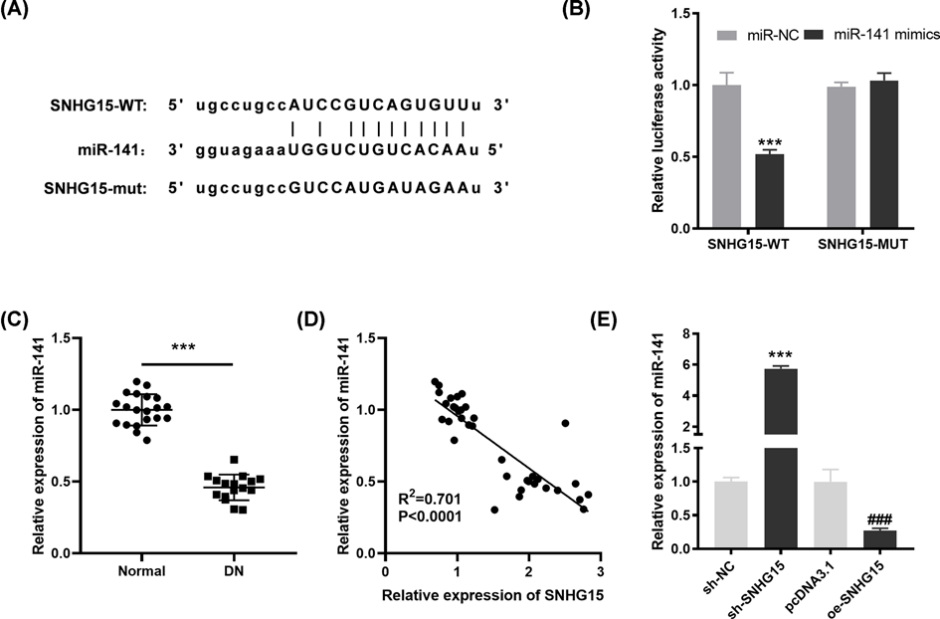
MicroRNA (MiR)-141 is the direct target of small nucleolar RNA host gene 15 (SNHG15) (**A**) The predicted complementary binding site of SNHG15 and miR-141. (**B**) The luciferase activity in human glomerular mesangial cells (HGMCs) co-transfected with pGL3-SNHG15 WT/pGL3-SNHG15 MUT and miR-141 mimics/NC was determined by dual luciferase reporter (DLR) assay. ***P<0.001 vs*.* the miR-negative control (NC) group. (**C**) The expression of miR-141 in diabetic nephropathy (DN) tissues (*n*=15) and normal tissues (*n*=20) was detected by quantitative reverse-transcription PCR (qRT-PCR). ***P<0.001 vs*.* normal. (**D**) The correlation between SNHG15 and miR-141; *P*<0.0001, *R*^2^ = 0.701. (**E**) The expression of miR-141 after transfection of shRNA (sh)-SNHG15/NC or overexpression (oe)-SNHG15/pcDNA3.1 into high-glucose (HG)-induced human glomerular mesangial cells (HGMCs) was detected by qRT-PCR. ***P<0.001 vs*.* the sh-NC group; ### *P*<0.001 vs*.* the pcDNA3.1 group.

Through Starbase software, a potential binding site between lncRNA SNHG15 and miR-141 was depicted (Figure 2A). DLR assay further demonstrated that in comparison with the SNHG15-WT/miR-NC group, the luciferase activity in the SNHG15-WT/miR-141 mimic group was obviously diminished (Figure 2B, *P*<0.001). Next, we utilized qRT-PCR to detect the expression of miR-141 in pediatric DN tissues and the results presented that miR-141 expression was down-regulated in pediatric DN tissues in contrast with the normal tissues (Figure 2C, *P*<0.001). Pearson’s correlation analysis uncovered that there was an inverse correlation between the expression of SNHG15 and miR-141 in pediatric DN tissues (Figure 2D; *P*<0.0001, *R*^2^ = 0.701). To further verify the correlation between SNHG15 and miR-141, miR-141 expression was detected after transfection of sh-SNHG15/NC or pDNA3.1/oe-SNHG15 into HG-stimulated HGMCs. The results of qRT-PCR displayed that miR-141 expression was up-regulated by SNHG15 knockdown and was down-regulated by SNHG15 overexpression (Figure 2E, *P*<0.001). The above data implied that miR-141 was the target of SNHG15 and was negatively modulated by SNHG1.

### Overexpression of miR-141 declines the levels of inflammatory factors in HG-induced HGMCs

**Figure 3:**
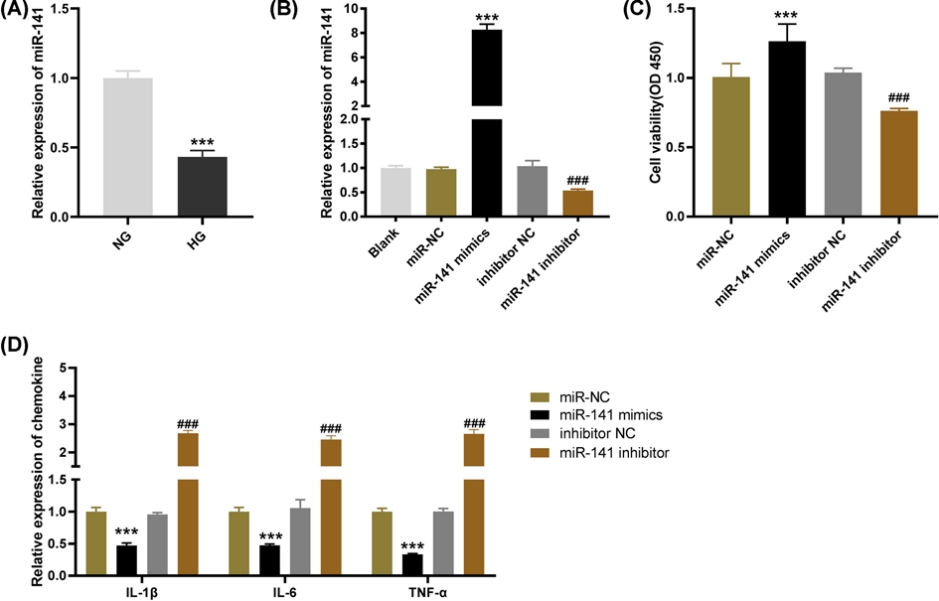
Overexpression of microRNA (miR)-141 declines the levels of inflammatory factors in high-glucose (HG)-induced human glomerular mesangial cells (HGMCs) (**A**) The expression of miR-141 in HG-stimulated HGMCs was determined by quantitative reverse-transcription PCR (qRT-PCR); ***P<0.001 vs*.* the normal glucose (NG) group. (**B**) The expression of SNHG15 after transfection of miR-141 mimics/negative control (NC) or miR-141 inhibitor/inhibitor NC into HG-stimulated HGMCs was detected by qRT-PCR. (**C**) The viability of HG-stimulated HGMCs was measured by MTT assay. (**D**) The levels of IL-1β, IL-6 and TNF-α in HG-stimulated HGMCs were measured by ELISA. ***P<0.001 vs*.* the miR-NC group; ### *P*<0.001 vs*.* the inhibitor NC group.

To further explore the biological function of miR-141 on pediatric DN *in vitro*, we first detected the expression of miR-141 in HG-induced HGMCs. The results of qRT-PCR demonstrated that miR-141 expression was dramatically reduced in HG-stimulated HGMCs in comparison with that of NG-treated cells (Figure 3A, *P*<0.001). Also, the efficiency of transfection was detected by qRT-PCR after transfection of miR-141 mimics/inhibitor into HG-stimulated HGMCs. We discovered that miR-141 expression was elevated by transfection of miR-141 mimics, whereas was declined by transfection of miR-141 inhibitor (Figure 3B, *P*<0.001), suggesting that miR-141 mimics/inhibitor was transfected into HG-induced HGMCs successfully. MTT assay displayed that transfection of miR-141 mimics significantly promoted cell viability while the viability of HG-induced HGMCs was suppressed by miR-141 inhibitor (Figure 3C, *P*<0.001). In addition, ELISA revealed that the levels of IL-6, IL-1β and TNF-α were decreased by miR-141 overexpression and increased by miR-141 down-regulation (Figure 3D, *P*<0.001). The above results indicated that overexpression of miR-141 could inhibit the viability and release of inflammatory factors in HG-induced HGMCs.

### MiR-141 targets ICAM-1

**Figure 4:**
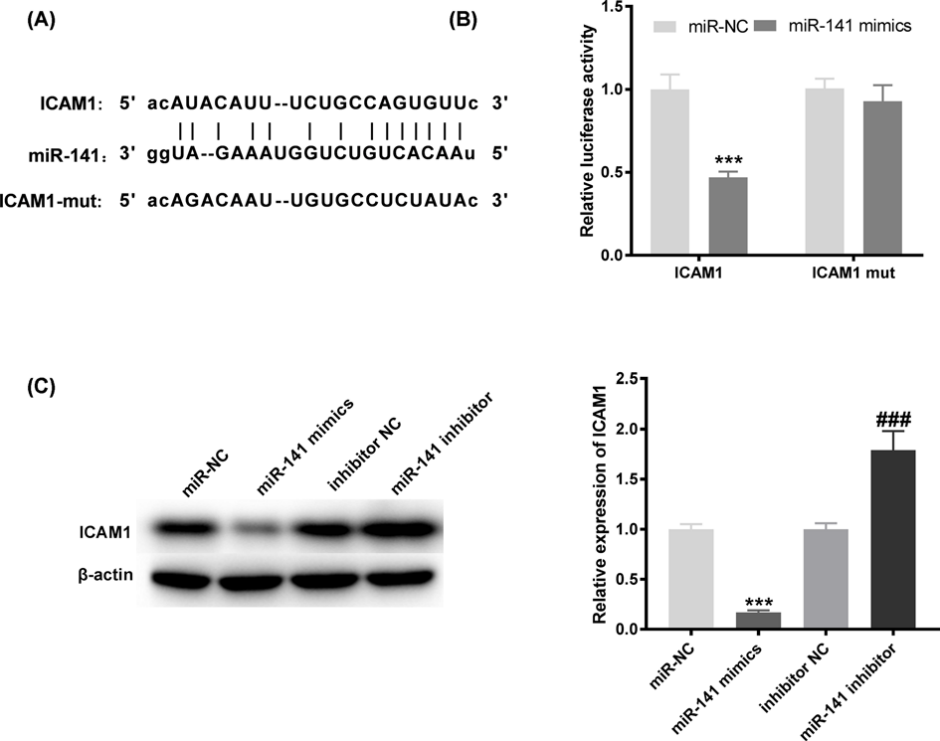
Intercellular adhesion molecule-1 (ICAM-1) is the target gene of microRNA (miR)-141 (**A**) The predicted complementary binding site of ICAM-1 and miR-141. (**B**) The luciferase activity in human glomerular mesangial cells (HGMCs) co-transfected with pGL3-ICAM-1 WT/pGL3-ICAM-1 MUT and miR-141 mimics/negative control (NC) was determined by dual luciferase reporter (DLR) assay. ***P<0.001 vs. the miR-NC group. (**C**) The protein level of ICAM-1 was measured by Western blot assay. ***P<0.001 vs. the miR-NC group; ###*P*<0.001 vs. the inhibitor NC group.

We predicted the potential binding site between ICAM-1 and miR-141 based on Targetscan software (Figure 4A). In contrast with the ICAM-1 WT/miR-NC group, a decreased luciferase activity in the ICAM-1-WT/miR-141 mimic group was exhibited by DLR assay (Figure 4B, *P*<0.001). To further verify the correlation between ICAM-1 and miR-141, Western blot was utilized to detect the protein level of ICAM-1 after transfection of miR-141 mimics/ inhibitor into HG-stimulated HGMCs. We found that ICAM-1 protein level was visibly inhibited by miR-141 mimics, while was elevated by miR-141 inhibitor (Figure 4C, *P*<0.001). The results suggested that ICAM-1 was the target gene of miR-141.

### MiR-141 diminishes the levels of inflammatory factors in HG-induced HGMCs through regulating ICAM-1

**Figure 5:**
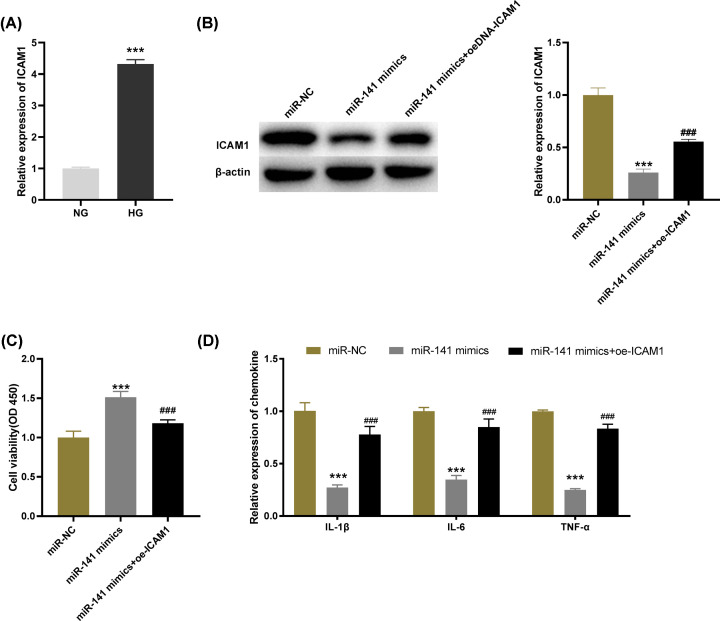
MicroRNA (MiR)-141 diminishes the levels of inflammatory factors in high-glucose (HG)-induced human glomerular mesangial cells (HGMCs) through regulating intercellular adhesion molecule-1 (ICAM-1) (**A**) The expression of ICAM-1 in HG-stimulated HGMCs was determined by quantitative reverse-transcription PCR (qRT-PCR); ***P<0.001 vs*.* the normal glucose (NG) group. (**B**) The protein level of ICAM-1 was measured by Western blot assay. (**C**) The viability of HG-stimulated HGMCs was measured by MTT assay. (**D**) The levels of IL-1β, IL-6 and TNF-α in HG-stimulated HGMCs were measured by ELISA; ***P<0.001 vs*.* the miR-negative control (NC) group; ### *P*<0.001 vs*.* the miR-141 mimics group.

qRT-PCR was utilized to detect ICAM-1 expression of HG-induced HGMCs and the results demonstrated that the expression of ICAM-1 was distinctly up-regulated in the HG group by contrast with the NG group (Figure 5A, *P*<0.001). Then based on Western blot analysis, we further determine the protein level of ICAM-1. The results revealed that up-regulation of ICAM-1 reversed the inhibitory effect of miR-141 mimics on ICAM-1 protein level (Figure 5B, *P*<0.001). Similarly, MTT assay and ELISA respectively illustrated that overexpression of ICAM-1 reversed the promoting effects of miR-141 mimics on cell viability and the suppressive effect on the levels of IL-6, IL-1β and TNF-α (Figure 5C,D, *P*<0.001). We speculated that the effects of miR-141 on cell viability and release of inflammatory factors were achieved through regulating ICAM-1.

### SNHG15 knockdown promotes the viability and inhibits inflammatory response of HG-induced HGMCs by regulating miR-141/ICAM-1

**Figure 6:**
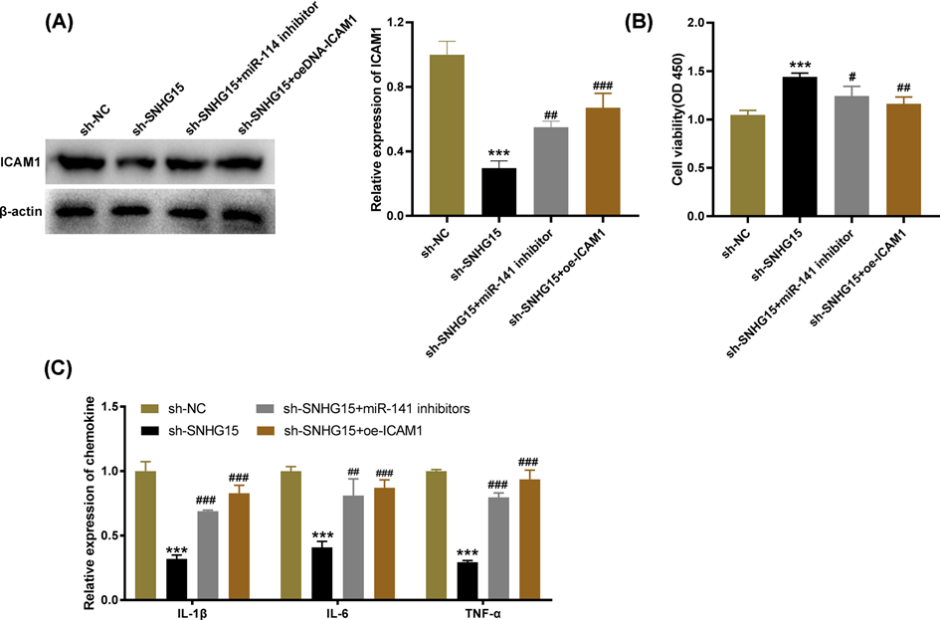
Small nucleolar RNA host gene 15 (SNHG15) knockdown promotes the viability and inhibits inflammatory response of high-glucose (HG)-induced human glomerular mesangial cells (HGMCs) by regulating microRNA (miR)-141/Intercellular adhesion molecule-1 (ICAM-1) (**A**) The protein level of ICAM-1 was measured by Western blot assay. (**B**) The viability of HG-stimulated HGMCs was measured by MTT assay. (**C**) The levels of IL-1β, IL-6 and TNF-α in HG-stimulated HGMCs were measured by ELISA. ***P<0.001 vs*.* the shRNA (sh)-negative control (NC) group; #*P*<0.05, ##*P*<0.01, ###*P*<0.001 vs*.* the sh-SNHG15 group.

To investigate the interactions among SNHG15, miR-141 and ICAM-1, the feedback verification experiments were performed. The results of Western blot presented that the protein level of ICAM-1 was declined by silencing of SNHG15. However, both down-regulation of miR-141 (*P*<0.01) and up-regulation of ICAM-1 (*P*<0.001) reversed the suppressive effect of SNHG15 knockdown on ICAM-1 protein level (Figure 6A, *P*<0.001). In addition, MTT assay and ELISA respectively uncovered that the low expression of miR-141 and high expression of ICAM-1 reversed the promoting effect of SNHG15 knockdown on cell viability and the inhibitory effects on levels of IL-6, IL-1β and TNF-α (Figure 6B,C, *P*<0.05). The above data indicated that SNHG15 knockdown might elevate the viability and suppress inflammatory response of HG-induced HGMCs by regulating the miR-141/ICAM-1 axis.

## Discussion

DN is one of the most renal diseases caused by hyperglycemia and inflammation, usually leading to serious damage of renal [24–26]. As previous studies mentioned, HG can damage cells by affecting diverse cellular processes, such as mitochondrial injury, oxidative damage and inflammation [27–29]. Because the pathological changes of HGMCs induced by HG are similar with the pathogenesis of DN, HG-induced HGMCs are commonly used to simulate the DN *in vitro* [27]. Therefore, an *in vitro* model of DN was established in HGMCs by HG treatment in this study. Researchers have displayed that lncRNAs play important roles in DN *in vivo* and/or *in vitro* [30–32]. Cheng et al. have uncovered that Dlx6os1 is significantly expressed in MCs under HG conditions by contrast with that in NG conditions [30]. Li et al. have illustrated that the increased MEG3 expression is observed in HG-stimulated MCs [31]. Also, Yang et al. have presented that XIST expression is up-regulated in DN tissues and HG-induced HK-2 cells [32]. Consistent with the above results, we disclosed that high expression of SNHG15 was exhibited in pediatric DN tissues and HG-induced HGMCs. Therefore, our data implied that SNHG15 might be a pathogenic lncRNA in DN.

Previous researches have been reported that aberrant expression of some lncRNAs is involved in modulating release of inflammatory factors in an *in vitro* model of DN [13,14,33]. For instance, silencing of Blnc1 visibly inhibited secretion of IL-6, IL-1β and TNF-α in HG-induced HK-2 cells [33]. In HG-induced MMCs, the expression of inflammatory cytokines was dampened by NEAT1 knockdown [14]. The levels of IL-6 and IL-1β in HG-treated MCs were restrained by down-regulation of NONHSAG053901 and elevated by up-regulation of NONHSAG053901 [13]. In the present study, we discovered that SNHG15 knockdown significantly declined the levels of IL-6, IL-1β and TNF-α, while overexpression of SNHG15 could promote secretion of IL-6, IL-1β and TNF-α in HG-induced HGMCs. The results indicated that silencing of SNHG15 could dampen the inflammatory response of DN *in vitro*. Similar to our results, a recent study has been illustrated that SNHG15 interference visibly suppresses the levels of inflammatory cytokines in mouse RAW264.7 cells [34]. However, this previous study only explored the effect of SNHG15 on inflammatory response in an *in vitro* model of spinal tuberculosis. Our results further verified that SNHG15 knockdown restrained inflammatory response of DN *in vitro*. Besides, in our research, we also found that the viability of HG-induced HGMCs was facilitated by transfection of sh-SNHG15 and suppressed by transfection of oe-SNHG15. Therefore, we speculated that SNHG15 knockdown inhibited the development of DN *in vitro*. However, the expression fold of SNHG15 was not consistent with the change folds of different cell processes, such as cell viability and inflammation. Because the molecular mechanism is complex in cell processes, the effect of SNHG15 may be influenced by other regulatory factors, especially for the adverse changes. Numerous studies have been revealed that miRNAs serve as suppressors in the levels of inflammatory factors of multiple diseases [35–38], including in DN *in vitro* [20–22]. Zha et al. have demonstrated that the decreased expression of miR-181a dramatically elevates the levels of inflammatory factors in HG-treated RAW264.7 cells, while the expression of inflammatory factors is inhibited by miR-181a overexpression [20]. Li et al. have disclosed that miR-218 expression is down-regulated in DN tissues and HG-induced murine podocyte cells, whereas overexpression of miR-218 attenuates the inflammatory damage to HG-induced murine podocyte cells [21]. A study conducted by Wu et al. has also uncovered that a low expression of miR-485 is observed in HG-treated human mesangial cells, while miR-485 up-regulation dampens the inflammation of HG-induced cells [22]. Consistent with the above researches, our study presented that miR-141 expression was also down-regulated in pediatric DN tissues and HG-induced HGMCs. IL-6, IL-1β and TNF-α levels were all suppressed by miR-141 overexpression. Cell viability promoted by miR-141 overexpression was also verified. At the same time, a negatively regulatory relationship between SNHG15 and miR-141 was confirmed. We conjectured that silencing of SNHG15 might mitigate DN progression *in vitro* through regulating miR-141. The feedback verification experiment between SNHG15 and miR-141 was performed to further verify this assumption. The relevant experiment demonstrated that miR-141 down-regulation reversed the inhibiting effect of SNHG15 knockdown on inflammation and the promoting effect on cell viability. The above results indicated that knockdown of SNHG15 alleviated inflammatory response of DN *in vitro* through regulating miR-141.

Intercellular adhesion molecule-1 (ICAM-1) belonging to immunoglobulin superfamily is an inflammatory sign and contributes to the progression of DN [39–41]. ICAM-1 has been confirmed to distinctly up-regulate in DN *in vitro* [42–45]. In our study, we also found in HG-induced HGMCs, the elevated expression of ICAM-1 was detected, which was consistent with the previous studies [46,47]. The results implied that ICAM-1 might be a promotor in DN. At the same time, ICAM-1 was identified to be a target gene of miR-141 and was negatively regulated by miR-141. We conjectured that miR-141 was involved in regulating the levels of inflammatory factors via regulating ICAM-1. Our feedback verification experiments that overexpression of ICAM-1 reversed the suppressive effect of miR-141 on IL-6, IL-1β and TNF-α levels further confirmed this hypothesis. In line with our results, a recent study has reported that miR-141 restrains inflammation caused by myocardial ischemia-reperfusion injury through negative regulation of ICAM-1 [48]. Additionally, in the above studies, we have confirmed that the interaction between SNHG15 and miR-141 on DN *in vitro*. We further conjectured that SNHG15 knockdown attenuated the inflammation of DN through modulating the miR-141/ICAM-1 axis. The feedback verification experiments between SNHG15 and ICAM-1 uncovered that the inhibitory effects of SNHG15 on the levels of inflammatory cytokines were dramatically reversed by ICAM-1 overexpression. Taken together, all the data in our study suggested that SNHG15 knockdown dampened inflammatory response of HG-induced HGMCs by regulating the miR-141/ICAM-1 axis. Additionally, ICAM-1 has been reported to interact with many signaling pathways in human diseases, such as ICAM-1-PI3K/Akt/GSK-3β/GATA-6 pathway in atheroscherosis [49], ICAM-1-IL-6/AKT/STAT3/NF-κB pathway in chronic obstructive pulmonary disease [50], and ICAM-1-PGE2/EP1 pathway in oral cancer [51]. Notably, ICAM-1 can interact with the SphK1-S1P pathway [52] and NF-κB pathway [53] in DN. We speculated that the SNHG15/miR-141/ICAM-1 axis may also be involved in the progression of DN by regulating these signaling pathways. Further researches on relevant mechanisms still need to be studied.

In summary, the present study uncovered SNHG15, which acts as an endogenous sponge of miR-141 to affect the development of pediatric DN *in vitro*. Up-regulation of miR-141 down-regulates ICAM-1, inhibiting the inflammatory reaction of pediatric DN *in vitro*. The present study reveals that the SNHG15/miR-141/ICAM-1 axis is essential in pediatric DN progression, pointing to SNHG15 may be a new therapeutic target for DN. However, the present study did not confirm the detailed mechanism among them *in vivo*, which may be a limitation of the present study. Further experiments will be performed to elucidate these issues in the future.

## Data Availability

All data can be obtained by contacting the corresponding author.

## Abbreviations

DLR: dual luciferase reporter
DN: diabetic nephropathy
ELISA: enzyme-linked immunosorbent assay
HG: high glucose
HGMC: human glomerular mesangial cell
ICAM-1: Intercellular adhesion molecule-1
lncRNA: long non-coding RNA
MA: microalbuminuria
SNHG15: small nucleolar RNA host gene 15
